# Fluoroscopy-Guided Colonoscopic Transanastomotic Drainage of a Delayed Presacral Collection After Rectal Cancer Surgery: A Case Report

**DOI:** 10.7759/cureus.116252

**Published:** 2026-09-14

**Authors:** Mehdi Zouaoui, Mohamed Aymane Assili, Youssef Hnach, Mbarek Azouaoui, Nourdin Aqodad

**Affiliations:** 1 Department of Hepato-Gastroenterology, Mohammed VI University Hospital Center, Agadir, MAR; 2 Faculty of Medicine and Pharmacy of Agadir, Ibn Zohr University, Agadir, MAR; 3 Department of Hepato-Gastroenterology, Oued Eddahab Military Hospital, Agadir, MAR

**Keywords:** anastomotic leakage, endoscopic drainage, presacral collection, rectal cancer surgery, transanastomotic drainage

## Abstract

Anastomotic leakage after rectal resection may present months after surgery as a contained presacral collection. When an established defect communicates with the rectal lumen, endoscopic transanastomotic drainage can use that tract without creating a new transmural access route. We report a 54-year-old man who presented six months after anterior rectal resection with fever, pelvic pain, tenesmus, and purulent rectal discharge. Pelvic magnetic resonance imaging showed a multiloculated 6 × 5 × 2 cm presacral collection with a fistulous tract to the posterior colorectal anastomosis. Radial endoscopic ultrasonography measured a longest cavity dimension of 87.1 mm, and colonoscopy identified a draining posterior anastomotic defect. After intravenous antibiotic therapy was initiated, the defect was cannulated under colonoscopic and fluoroscopic guidance, the cavity was lavaged, and a 7-Fr × 7-cm double-pigtail plastic stent was deployed for internal drainage. Symptoms and inflammatory markers normalized within one week. At two months, a residual 40-mm cavity remained, and a second identical stent was placed alongside the first. At three months, the patient remained asymptomatic, but both stents were still in situ, and definitive cavity closure had not yet been documented. This case demonstrates the technical feasibility of transanastomotic drainage and short-term clinical control in a selected patient without peritoneal signs; it does not establish durable healing or generalizable efficacy.

## Introduction

Anastomotic leakage after colorectal surgery is a defect in intestinal-wall integrity at the anastomotic site that creates communication between the intra- and extraluminal compartments. The International Study Group of Rectal Cancer grades leakage by its effect on clinical management [1]. Although many leaks present early, delayed leakage after low anterior resection can evolve into a chronic presacral sinus or collection, and neoadjuvant treatment may increase the complexity of this problem [2].

Management is guided by clinical stability, the severity of sepsis, collection anatomy, and the availability of a safe access route. Options include antibiotics, percutaneous or surgical drainage, endoscopic ultrasound-guided drainage, and endoscopic vacuum therapy [3-6]. When a visible anastomotic defect communicates directly with the collection, endoscopic transanastomotic drainage can use the existing tract to provide internal drainage [7,8]. We describe the technique and short-term outcome in a patient with a delayed, contained presacral collection after rectal cancer surgery.

## Case presentation

Patient history and presentation

A 54-year-old man with mid-rectal adenocarcinoma received neoadjuvant chemoradiotherapy, followed by laparoscopic anterior rectal resection in April 2025. Six months after surgery, he presented with fever, pelvic pain, tenesmus, and purulent rectal discharge. His temperature was 38.5°C, his World Health Organization performance status was 1, and his body mass index was 22 kg/m². Abdominal examination showed hypogastric tenderness without guarding or rebound. Digital rectal examination identified purulent exudate. The clinical and laboratory findings at admission are summarized in Table 1.

**Table 1:** Available vital signs, clinical status, and initial laboratory findings at admission Note: Reference intervals are those used by the reporting institution. WHO, World Health Organization; kg/m², kilograms per square meter; g/dL, grams per deciliter; fL, femtoliters; x10^9^/L, 10^9^ cells per liter; mg/L, milligrams per liter; g/L, grams per liter; mmol/L, millimoles per liter

Parameter	Patient value	Unit	Reference range
Body temperature	38.5	degrees C	36.0-37.5
Body mass index	22	kg/m²	18.5-24.9
WHO performance status	1	0-5 scale	0 (fully active)
Hemoglobin	10.2	g/dL	13.0-17.0
Mean corpuscular volume	78	fL	80-100
Mean corpuscular hemoglobin concentration	30	g/dL	32-36
Platelet count	195	x10^9^/L	150-400
White blood cell count	18.5	x10^9^/L	4.0-10.0
Absolute neutrophil count	13.2	x10^9^/L	1.5-7.5
C-reactive protein	55	mg/L	<5
Urea	0.20	g/L	0.17-0.49
Serum creatinine	8	mg/L	6.7-11.7
Sodium	134	mmol/L	136-145
Potassium	3.8	mmol/L	3.5-5.1
Prothrombin activity	90	%	70-100
Serum albumin	30	g/L	34-50

Imaging and endoscopic assessment

Pelvic magnetic resonance imaging showed a multiloculated presacral collection measuring 6 × 5 × 2 cm and a fistulous tract communicating with the posterior aspect of the colorectal anastomosis (Figure 1).

**Figure 1:**
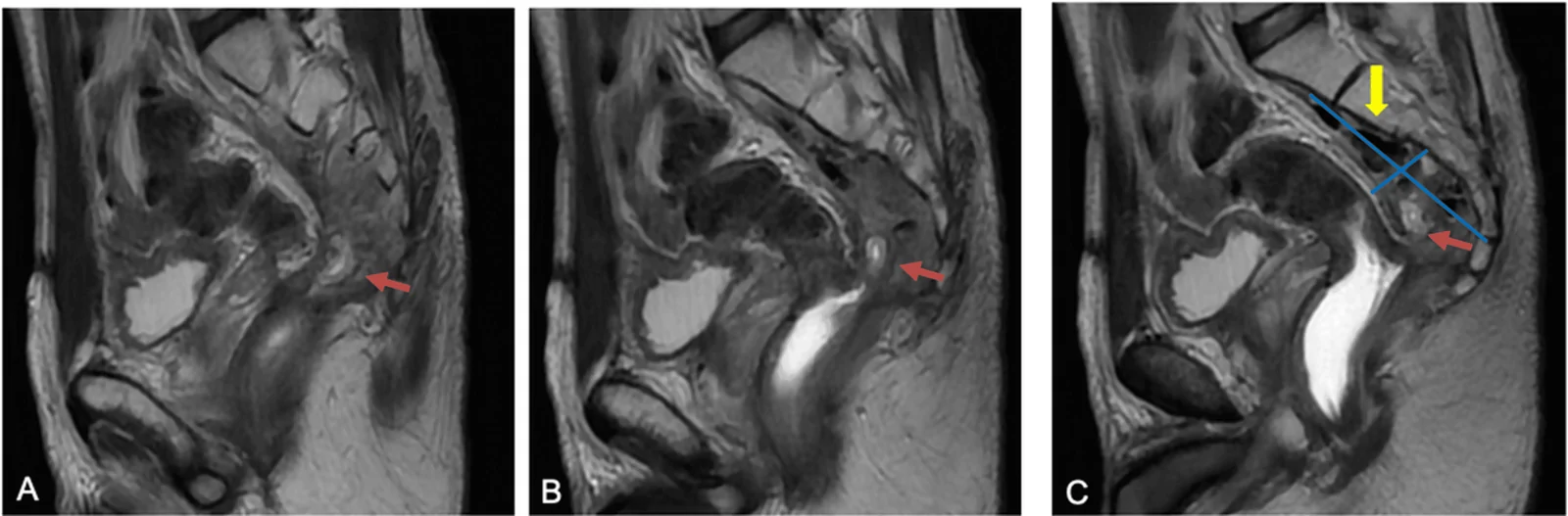
Sagittal T2-weighted pelvic magnetic resonance images without fat suppression (A-C) The red arrows indicate the fistulous tract extending from the posterior aspect of the colorectal anastomosis into the presacral space. The yellow arrow in panel C indicates the presacral collection, and the blue lines delimit its measured extent.

Radial endoscopic ultrasonography performed approximately 5 cm from the anal verge showed a heterogeneous cavity with a longest endosonographic dimension of 87.1 mm (Figure 2). This was a single-plane endosonographic measurement rather than a repeat three-axis magnetic resonance measurement. Endoscopic inspection demonstrated a posterior anastomotic defect with spontaneous purulent drainage, providing direct access to the cavity.

**Figure 2:**
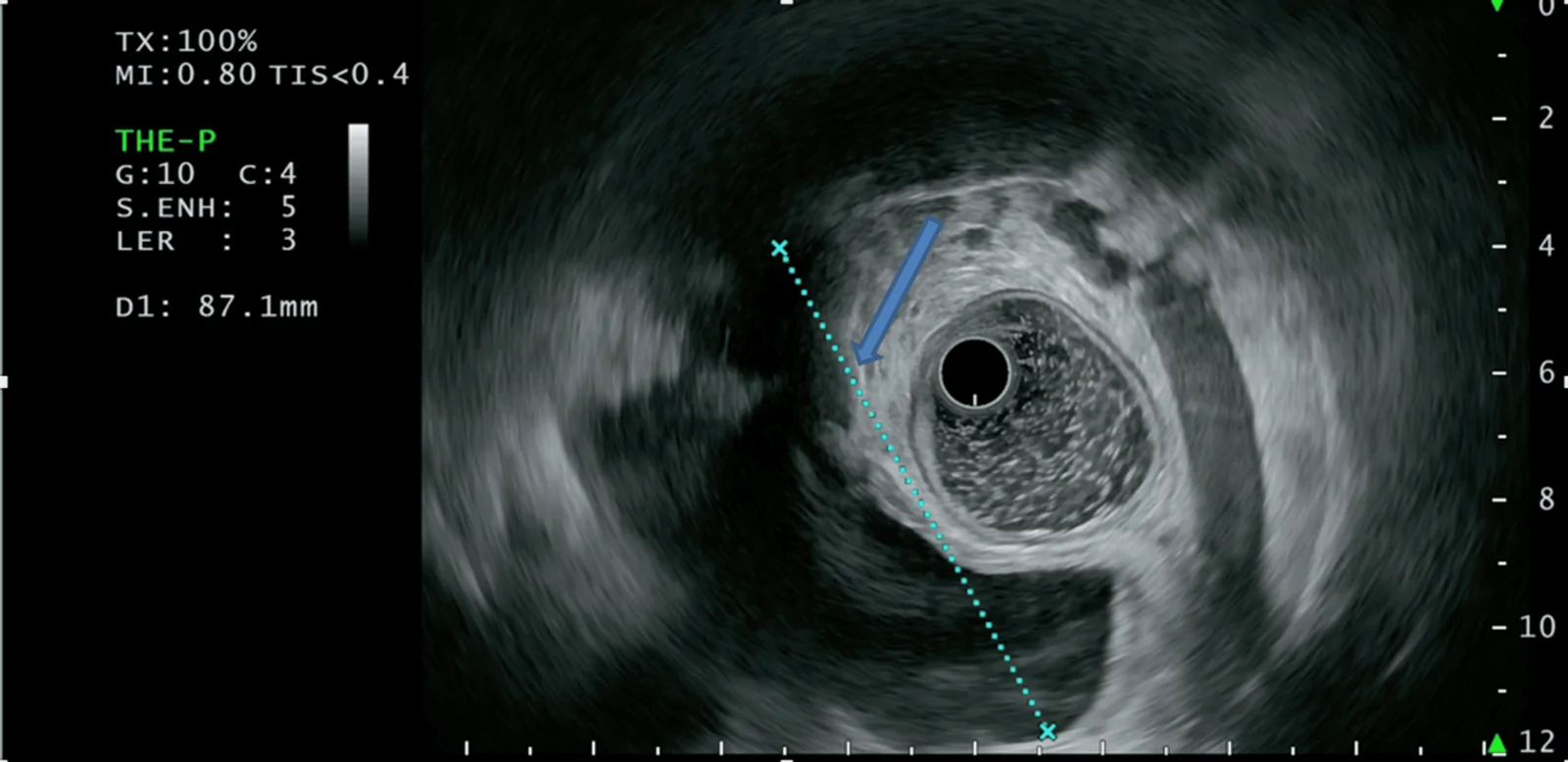
Baseline radial endoscopic ultrasonography demonstrating a heterogeneous presacral collection (blue arrow) with a maximal measured diameter of 87.1 mm, shown by the dotted caliper line

Endoscopic transanastomotic drainage 

The patient received systemic ceftriaxone 2 g/day and metronidazole 1.5 g/day. The drainage procedure was performed with an Olympus EVIS X1 CV-1500 platform and a CF-HQ190L therapeutic colonoscope. Direct endoscopic inspection identified the posterior fistulous opening at the anastomosis, approximately 5 cm from the anal verge. Purulent material drained through the opening. The tract was selectively cannulated with a 7-Fr, 200-cm tapered drainage catheter and a 0.035-inch, 450-cm hydrophilic guidewire (Figure 3).

**Figure 3:**
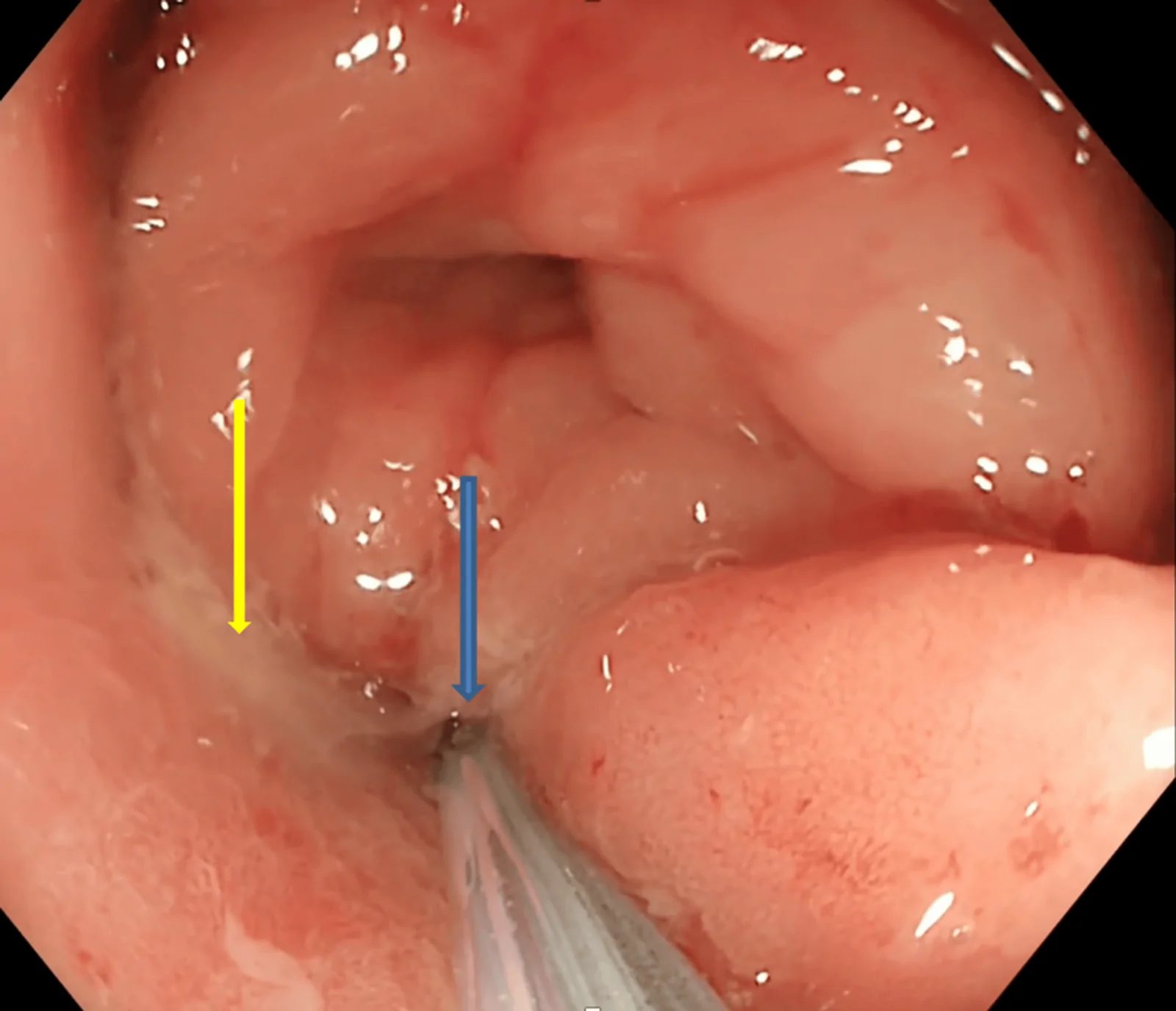
Direct colonoscopic view of the posterior anastomotic fistulous opening The blue arrow indicates the catheterized tract, and the yellow arrow indicates purulent drainage.

Under fluoroscopic control, the guidewire was advanced and coiled within the collection, confirming stable intracavitary access (Figure 4). The cavity was then extensively irrigated with isotonic saline. One ampoule of gentamicin containing 80 mg in 2 mL was diluted with 9 mL of isotonic saline and instilled into the cavity, where it was left as an adjunct to mechanical drainage and systemic antibiotic therapy.

**Figure 4:**
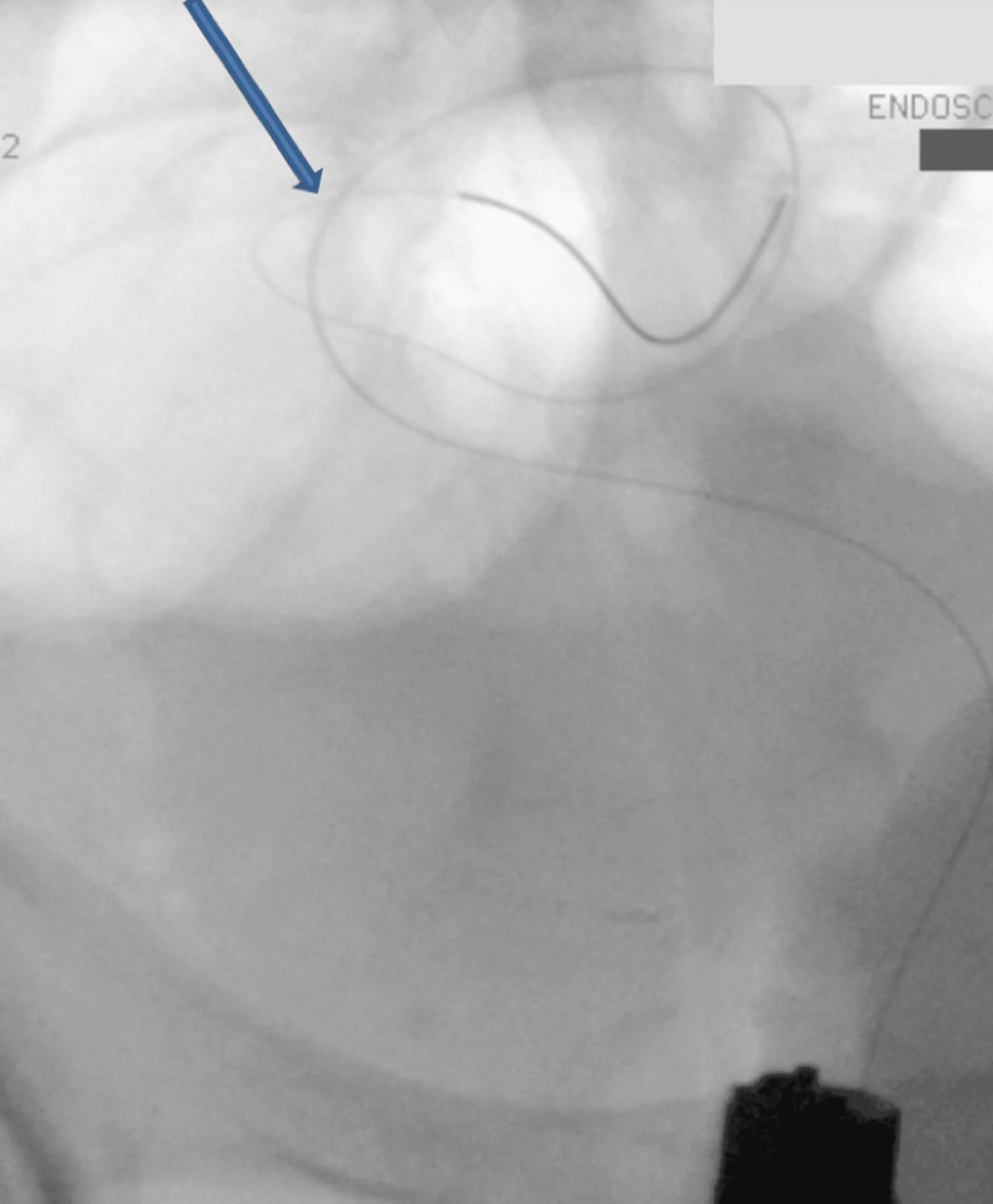
Fluoroscopic image demonstrating coiling of the hydrophilic guidewire within the presacral collection (blue arrow), confirming intracavitary access before stent deployment

A 7-Fr x 7-cm double-pigtail plastic stent was subsequently deployed over the guidewire. Fluoroscopic and endoscopic controls confirmed that the proximal pigtail was positioned within the presacral collection and that the distal pigtail remained within the rectal lumen at approximately 5 cm from the anal verge, with continued purulent drainage (Figure 5).

**Figure 5:**
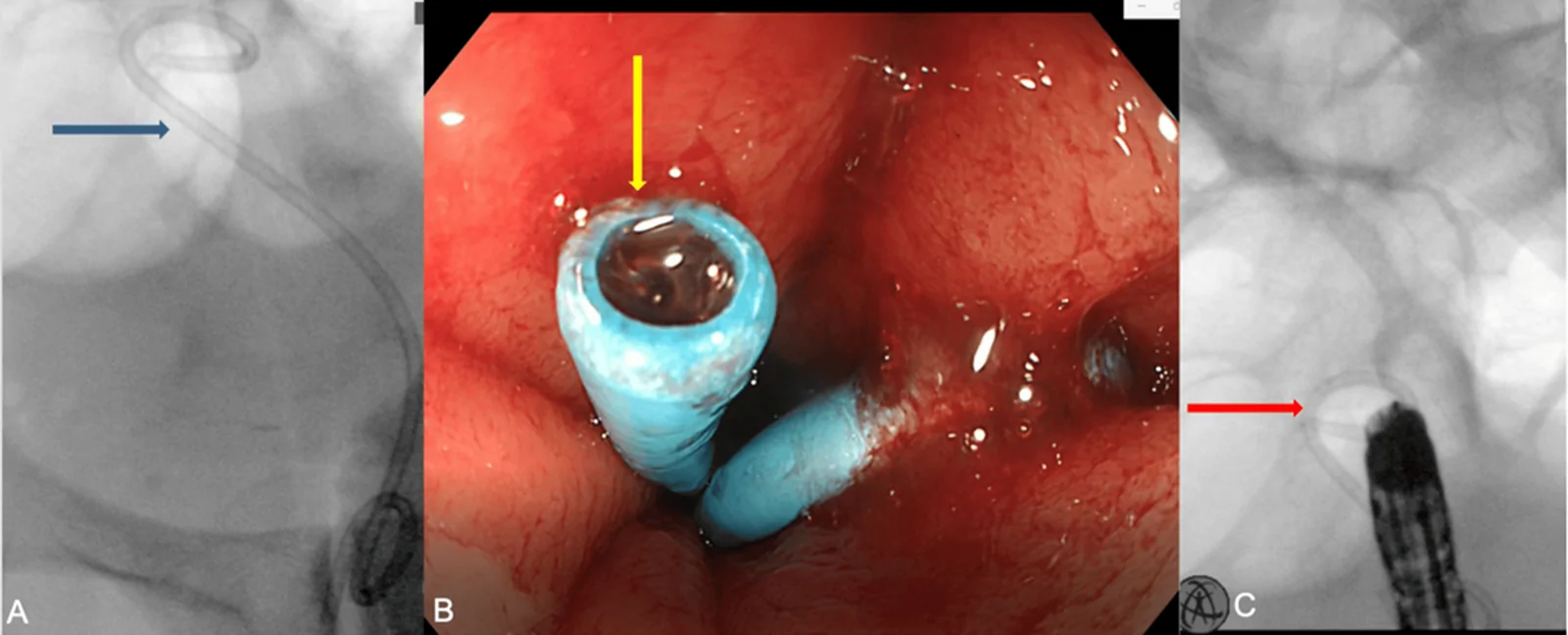
Post-deployment assessment of the first double-pigtail stent (A) Fluoroscopic overview showing the stent in position (blue arrow). (B) Endoscopic view of the distal pigtail within the rectal lumen approximately 5 cm from the anal verge, with purulent drainage through the stent (yellow arrow). (C) Fluoroscopic view confirming the proximal pigtail within the presacral collection (red arrow).

Clinical course and follow-up

Fever, pelvic pain, tenesmus, and purulent discharge improved rapidly after drainage. At one week, the patient was afebrile and inflammatory markers had normalized. At two months, radial endoscopic ultrasonography showed a residual cavity measuring approximately 40 mm; the first stent remained patent. A second 7-Fr × 7-cm double-pigtail plastic stent was placed alongside the first to augment internal drainage (Figure 6).

**Figure 6:**
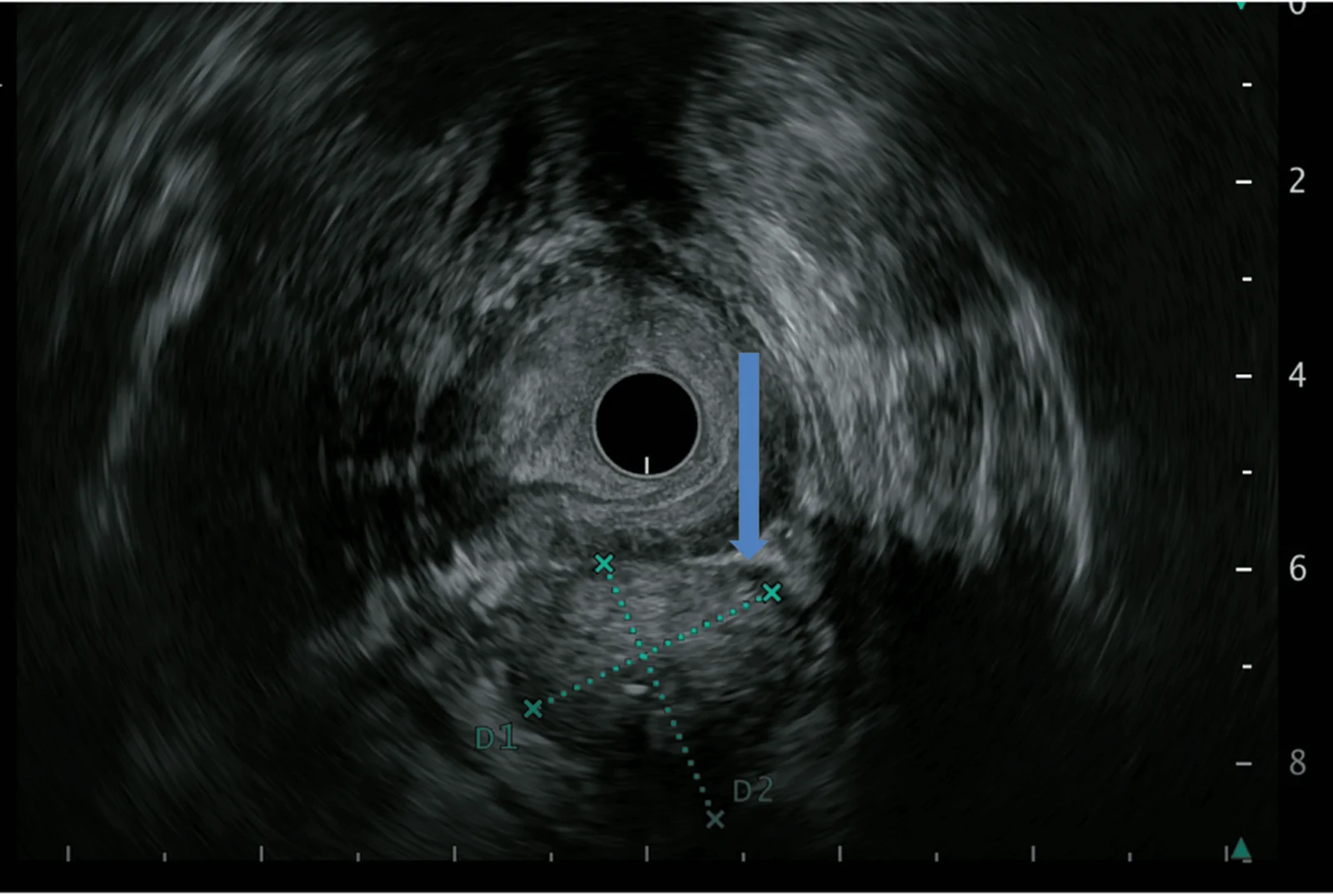
Radial endoscopic ultrasonography two months after the initial drainage showing a residual presacral collection measuring approximately 4 cm in maximal diameter (blue arrow and dotted calipers)

At three months after the initial drainage procedure, the patient remained asymptomatic and afebrile, and inflammatory markers remained normal. Both stents were still in situ. Further endoscopic and imaging follow-up was arranged to document cavity closure and determine the timing of stent removal.

## Discussion

This presentation is compatible with grade B anastomotic leakage because it required active intervention without relaparotomy [1]. Its occurrence six months after surgery illustrates the delayed end of the leakage spectrum and the potential for a leak to persist as a presacral sinus or collection [2]. The prompt symptomatic and inflammatory improvement in this patient represents short-term clinical control. The residual cavity at two months and the presence of both stents at three months preclude a claim of definitive healing.

Stable patients with a contained pelvic collection may be candidates for minimally invasive drainage, whereas generalized peritonitis, uncontrolled sepsis, tissue ischemia, or the absence of a safe access route generally favors surgical management. Endoscopic ultrasound-guided pelvic drainage has shown favorable technical and clinical outcomes in systematic and multicenter reports [3-5]. Endoscopic vacuum therapy is another organ-preserving option, although it commonly requires repeated endoscopic exchanges and suitable access to the cavity [6].

Transanastomotic drainage differs from transmural endoscopic ultrasound-guided access because it uses a pre-existing anastomotic opening. In a 14-patient pilot study, Blot et al. reported successful stent placement in 13 patients (92.8%) and overall treatment success in 11 patients (78.5%) [7]. In a later 12-patient case series, Khan et al. reported management without anastomotic revision or a new diverting stoma in all patients [8]. These uncontrolled series involve selected patients, varied techniques, and limited sample sizes; they do not permit direct comparison with other drainage strategies.

In the present case, the visible posterior defect permitted catheter and guidewire access under fluoroscopy, and the double-pigtail stent maintained internal drainage. Placement of a second stent for the residual cavity was a case-specific decision rather than an established staged multi-stent protocol. The intracavitary gentamicin instillation was also operator-selected and unvalidated. Its effect cannot be separated from that of systemic antibiotics, saline lavage, and mechanical drainage.

This report is limited by its single-patient design, absence of a comparator, and short follow-up. At the last assessment, stent removal and definitive radiologic or endoscopic closure had not been documented. Accordingly, the case contributes a technical description and a signal of short-term feasibility rather than evidence of durable efficacy. Longer follow-up and comparative studies are needed to define patient selection, optimal stent number and dwell time, and recurrence rates.

## Conclusions

Direct transanastomotic drainage through a visible colorectal anastomotic defect was technically feasible in this stable patient with a contained delayed presacral collection. Internal double-pigtail stenting was associated with prompt clinical and inflammatory improvement, while a residual cavity required a second stent at two months. Because the stents remained in place and definitive closure had not been documented at three months, the outcome should be considered short-term control rather than cure. Patient selection, procedural expertise, and structured imaging and endoscopic follow-up are essential, and comparative evidence is needed before routine adoption.
